# Salting-out assisted liquid-liquid extraction utilizing binary salts for the simultaneous determination of chloro-*s*-triazines and their major degradation products in environmental water and fruit samples by HPLC-DAD

**DOI:** 10.1186/s13065-026-01767-4

**Published:** 2026-03-01

**Authors:** Tura Gemechu, Abi Legesse, Negussie Megersa, Bhagwan Singh Chandravanshi

**Affiliations:** 1 Department of Chemistry, College of Natural and Computational Sciences, Addis Ababa University, P. O. Box 1176, Addis Ababa, Ethiopia; 2 Department of Chemistry, College of Natural and Computational Sciences, Ambo University, P.O. Box 19, Ambo, Ethiopia

**Keywords:** Multi-residue, SALLE, Metabolites, Fruit juices, Water samples, Chloro-*s*-triazine pesticides

## Abstract

**Background:**

Symmetrical (*s-*) triazine herbicides are the most commonly used pesticides, which frequently leave pesticide residues in food and environmental samples. Due to their carcinogenic and endocrine-disrupting properties, monitoring *s*-triazine herbicide residues in the water and food samples has become a critical concern of environmental and public health protection. Furthermore, there is no reported study on the application of salting-out assisted liquid-liquid extraction (SALLE) for simultaneous extraction of chloro-*s*-triazines and their major degradation products from environmental water and fruit samples. Therefore, SALLE method was developed for the simultaneous extraction of chloro-*s*-triazine pesticides and their degradation products from diverse samples by HPLC-DAD.

**Methods:**

In the present study, SALLE method based on binary salts was developed for the simultaneous extraction of multi-residue chloro-*s*-triazine pesticides, including (atrazine, cyanazine, and simazine), with their degradation products (deethylatrazine and deisopropylatrazine) from environmental water and fruit samples prior to analysis by HPLC-DAD. Various parameters that can affect the method, such as extraction solvent type and volume, type, mass and ratio of salts and pH effect, centrifugation and vortexing times were successfully optimized.

**Results:**

The developed method provided good linearity with coefficients of regression in the range of 0.991–0.997, under the optimized conditions. The method demonstrated lower limit of detection and limit of quantification in the range of 0.002–0.089 and 0.0066–0.299 µg/L, respectively. Repeatability and reproducibility in terms of %RSD were found in the range of 2.15–7.13% and 3.28–8.95%, respectively. The mean recovery was varied from 74.06 to 109.90% with RSD (*n* = 3) below 10% for all samples studied.

**Conclusion:**

The developed method has been successfully applied for the trace level extraction of pesticide residues and their degradation products in real samples, including environmental waters, and fruit samples. Hence, the developed method can be used as a selective and green alternative for the extraction of pesticide residues in water, fruits and other contaminated food samples.

## Background

Pesticides are chemical compounds commonly used to protect crops, livestock, and human environments from various pests. Although they contribute to increase agricultural yields, their residues frequently remain in the environment, contaminating the soils, waters, and food sources [1]. In developing nations such as Ethiopia, pesticide application has increased alongside agricultural expansion and reform efforts through years. However, only less than 0.1% of the applied pesticides actually hit the intended targets, while the majority disperses into the environment and accumulate in unintended locations [2, 3].

Symmetrical (*s-*) triazine herbicides, such as atrazine, cyanazine and simazine, are among the most commonly used pesticides, which frequently leave detectable pesticide residues in food and environmental samples. In particular, the major degradation products, including deethylatrazine and deisopropylatrazine are also frequently found in various environmental compartments [4–7]. The rate of degradation of atrazine in soil is heavily influenced by natural and environmental factors including temperature and rainfall, making residue-level monitoring very essential. These herbicides and their breakdown products pose contamination risks not only to crops but also to the surrounding soil and natural water sources [8]. They function by disrupting photosynthesis in broad-leaved weeds, but their environmental release allows residues to enter human body through the food chain, where they can accumulate over times [9, 10]. Exposure to these compounds has been linked to adverse health effects, including skin irritations, hormonal imbalances, birth defects, and certain types of cancers [11].

Due to their carcinogenic and endocrine-disrupting properties, even small quantities of these residues raise significant concerns for environmental and public health. As a result, monitoring *s*-triazine herbicide residues in water and food samples has become a critical priority. Additionally, monitoring their degradation products regularly and frequently is equally important, as some metabolites could be even more toxic than the parent compounds [12]. Consequently, there is a demanding need for development of sensitive, simple, rapid, and environmentally friendly analytical methods to detect and quantify the residues of the *s*-triazine herbicides and their degradation products. In most cases, the extraction and preconcentration steps are often more critical than the final determination due to the complex nature of the matrices and the availability of analytes at trace levels [13].

Numerous sample preparation methods have been developed and used for the extraction and preconcentration of these herbicide residues and their degradation products from complex environmental, biological, food and various water resources. Traditional extraction methods, including liquid-liquid extraction (LLE) [14, 15] and solid-phase extraction (SPE) [16], have been widely used but suffer from a number of drawbacks such as labor-intensive procedures, long processing times, low enrichment factor, and the use of large volumes of toxic organic solvents, which limit their extensive applications. Solid phase microextraction (SPME) [17–19] has emerged as a rapid and solvent-free alternative, allowing simultaneous extraction and preconcentration of pesticide residues from different matrices. However, its high cost, fiber fragility, and limited lifespan restrict its broader application.

Liquid-phase microextraction (LPME) offers a miniaturized and greener alternative to conventional LLE, overcoming many of its limitations while addressing some of the SPME’s shortcomings [20, 21]. Various LPME methods, such as hollow-fiber liquid phase microextraction (HF-LPME) [22], dispersive liquid-liquid microextraction (DLLME) [23, 24] and single-drop microextraction (SDME) [25] have been widely utilized for the extraction of pesticides from different matrices. The methods use minimal organic solvents, ensuring high enrichment factors [26]. DLLME in particular is one of the most commonly employed methods for its high efficiency, speed, simplicity, and minimal organic solvent consumption [27]. It is frequently applied to analyze trace analytes in food, environmental waters, and biological samples [28]. Despite the undeniable advantage of DLLME, it has a potential loss for analyte during cleanup, reduced partitioning for some pesticides due to disperser solvents, and challenges with highly polar/hydrophilic pesticides, requiring careful solvent selection [29].

Another promising method for the extraction of pesticides is salting-out assisted liquid-liquid extraction (SALLE), which employs extraction solvents like acetonitrile, acetone, or isopropanol alongside salt-induced phase separation [20]. This technique involves addition of electrolyte solutions to the sample solution to enhance the distribution ratio of a specific solute. Addition of salt reduces interfacial tension between the aqueous phase and the extractant, thereby improving extraction efficiency through better solvent dispersion. SALLE is recognized for its simplicity, cost-effectiveness, and environmental friendliness. It combines extraction, clean-up, and preconcentration into a single step, merging the advantages of QuEChERS [30]. The method has been successfully applied for the extraction of pesticides in foods, biological matrices, and environmental waters [31]. Despite its advantages, SALLE has been rarely employed for the extraction and enrichment of chloro-s-triazine pesticides, and their degradation products in environmental water and food samples.

Notably, there is no reported study on the application of SALLE for simultaneous extraction of chloro-*s*-triazines and their major degradation products from environmental water and fruit samples. This study, therefore, designed to optimize, validate and develop simple, fast, eco-friendly and cost-effective SALLE method, utilizing binary salt solution, for the preconcentration, enrichment and quantification of atrazine (ATZN), simazine (SMZN), cyanazine (CYZN), deethylatrazine (DEA), and deisopropylatrazine (DIA) in water samples (river, lake and ground water) and fruit sample juices (pineapple, orange, and watermelon), followed by HPLC-DAD analysis. Key experimental parameters were studied to establish optimum conditions. The method’s performance was validated and the results were compared with those reported in literature. Finally, the method’s applicability was assessed by extracting pesticide residues and their metabolites in the real samples.

## Materials and methods

### Chemicals and reagents

Pesticide standards utilized in the present experiments were of analytical grade reagents; viz., ATZN, SMZN, CYZN, DEA, and DIA which were obtained from Sigma-Aldrich (Steinheim, Germany). All these standards were of high purity, i.e., > 98%. Other types of common chemicals utilized in this study were also analytical-grade reagents while the solvents utilized, such as ethyl acetate, acetonitrile (ACN), acetone, and methanol acquired from Sigma-Aldrich (Steinheim, Germany), were HPLC-grade solvents. Magnesium sulfate (MgSO_4_) and ammonium sulfate ((NH_4_)_2_SO_4_) were obtained from Fine Chem Industries (Mumbai, India, 99%). Ammonium carbonate ((NH_4_)_2_CO_3_) and sodium chloride (NaCl), obtained from VWR International (Radnor, PA, USA) were used for salting out optimization in the experiment. Common chemicals such as sodium hydroxide (NaOH) (Merck chemicals, Darmstadt, Germany) and hydrochloric acid (HCl) (Sigma-Aldrich, St. Louis, MO, USA), were utilized for pH adjustments. A double distiller (800 Aquatron, Bibby Scientific, Staffordshire, UK) and deionizer (Easy Pure LF, Dubuque) were used to prepare and purify ultrapure water.

### Instruments and equipment

Chromatographic determinations were conducted using Agilent 1200 series HPLC system (Agilent Technologies, Waldbronn, Germany), which included a vacuum degasser, a quaternary pump, a standard and preparative autosampler with a thermostat, thermostated column compartment, and a diode array multiple wavelength detector. Data acquisition and sample processing were managed using LC Chemstation software (B.02, 01-SR1). Separation was performed with analytical ZORBAX Octadecylsilane (ODS - C18, 150 × 3 mm, i.d., 3.5 μm particle size) column from Agilent Technologies. A pH meter (Adwa, model 1020, Romania) was used to measure the pH of the solution. For sample preparation, a centrifuge (Model 80 − 2, Jiangsu Zhenji Instruments Ltd., Jiangsu, China), 15 mL centrifuge tubes (Corning integrated, Corning, NY, Mexico) and a vortex mixer (Griffin and George Ltd, Britain) were utilized. Sample solutions were filtered using a 0.20 μm micro-syringe filter (Sartorius Stedim Biotech GmbH, Goettingen, Germany).

### Preparation of standard solutions

Sample solutions of the analytes were prepared based on the previous work published from our research group with modifications [1]. Accordingly, a stock solution of each target standard was prepared at concentration of 100 µg/mL, by accurately weighing the required amounts and dissolving in methanol. Next, a 10 µg/mL intermediate solution was then prepared by diluting the stock solution using ultrapure water. Additional working solutions at lower concentrations were then prepared from the intermediate solution using ultrapure water. All standard solutions were stored in a refrigerator at temperatures less than 4 °C when not in use. Table 1 provides the physicochemical properties of the standards utilized in this study.

**Table 1:** Structure and physicochemical properties of the pesticide standards utilized in the study

Common name	IUPAC name		Structure	Solubility (mg/L) at 25 °C)	log K_ow_ (25 °C)	pK_a_ (25 °C)
Atrazine	1-Chloro-3-ethylamino-5-isopropylamino-2,4,6-triazine			33	2.50	1.70
Deethylatrazine	6-Chloro-N-isopropyl-1,3,5-triazine-2,4-diamine			8.6	1.52	1.30
Deisopropylatrazine	6-Chloro-N-2-ethyl-1,3,5-triazine-2,4-diamine			6.2	1.15	1.30
Cyanazine	2-{[4-Chloro-6-(ethylamino)−1,3,5-triazin-2-yl]amino}−2-methylpropane nitrile			171	2.2	1.0
Simazine	6-Chloro-N-2,4-diethyl-1,3,5-triazine-2,4-diamine			34.7	2.1	1.62

K_ow_: octanol–water partition coefficient

pK_a_: acidity constant

### Chromatographic conditions

Chromatographic separation was achieved using an isocratic solvent delivery mode with a tertiary mobile phase composition, consisting 48% water (ultrapure), 22% acetonitrile, and 30% methanol. The column of HPLC was conditioned and equilibrated with the optimized mobile phase for about 10 min, prior to the sample injection. The analysis was carried out at 0.7 mL/min flow rate, 35 °C column temperature, 10 µL volume of injection, and 224 nm UV detection wavelength for all the analytes under the study. The peak areas were used as chromatographic response in parameter optimization and quantitative analysis. Under these experimental conditions, good base-line separation was achieved for all target pesticide analytes in the study.

### Sample collection and preparation of fruit and water samples

Water and fruit samples were collected from different areas. River, lake, and groundwater samples were taken from Bishoftu city, 50 km east of Addis Ababa; the capital of Ethiopia, geographically located at 8°44′40″ N latitude and 38°59′9″ E longitude, with altitude of approximately 1,920 m above sea level. The water samples were collected in polypropylene bottles, each labeled with codes: RW, GW and LW for river, ground, and lake water, respectively. After collection, the water samples were transported to the Analytical Chemistry Research Laboratory at Addis Ababa University. Prior to SALLE extraction, all the environmental water samples were filtered through a 0.45 μm micropore membrane filter.

Pineapple, watermelon and orange fruit samples were collected from local supermarkets in Addis Ababa, located at, approximately, 8°59′ N latitude and 38°48′ E longitude, with an altitude of about 2,355 m above sea level. Each type of collected fruit was homogenized separately, utilizing a mixer, and then filtered through Whatman number 42 filter paper. The residues on the filter paper were washed multiple times with 1 mL of ultrapure water to ensure complete extraction into the filtrate. The prepared juice samples were then centrifuged at 4,000 rpm for 5 min. The supernatant was diluted at a 2:1 volume ratio with ultrapure water, and subsequently filtered through a 0.20 μm micro-syringe filter before undergoing the extraction procedure. The extracted fruit juice and water samples were stored in a refrigerator at 4 °C until further analysis.

### SALLE extraction procedure

Aliquots of 0.5 mL of freshly prepared fruit juice samples were placed in 15 mL falcon tubes, and diluted to 5.0 mL with ultra-pure water (pH 7.0) to minimize the effect of matrix. The pH of the solution was adjusted by using NaOH or HCl (0.1 M), and appropriate amount of a pesticide standard mixture was added. The spiked solution was allowed to stand for approximately 10 min to equilibrate. Then, 2 mL of ACN was added, and the mixture was vortexed for 1 min. This was followed by addition of 2 g MgSO_4_ and vortexing the mixture for additional two minutes to dissolve the salt, which acts as a salting out agent. After centrifuging the mixture for 5 min at 4000 rpm, 500 µL of the supernatant was carefully taken with a micropipette and transferred to the injection vial. Finally, 10.0 µL of the supernatant was injected into the HPLC–DAD system for analysis. A similar extraction procedure was followed for water samples except for the final dilution of the resulting extract with ultrapure water.

## Results and discussions

### SALLE procedure for trace level pesticide extraction

During method development, various extraction parameters have been optimized; including type and volume of the organic solvent, type and amount of the salts, sample solution pH, agitation time, and vortex time. All experimental parameters were optimized in triplicate, and each extract solution was injected twice for duplicate analyses. The peak areas of the target analytes served as the instrumental response, enabling assessment of the impact of these parameters on the SALLE, and establish optimum experimental conditions for all the studied factors.

### Optimization of the extraction parameters

#### Selection of extraction solvent

**Fig. 1:**
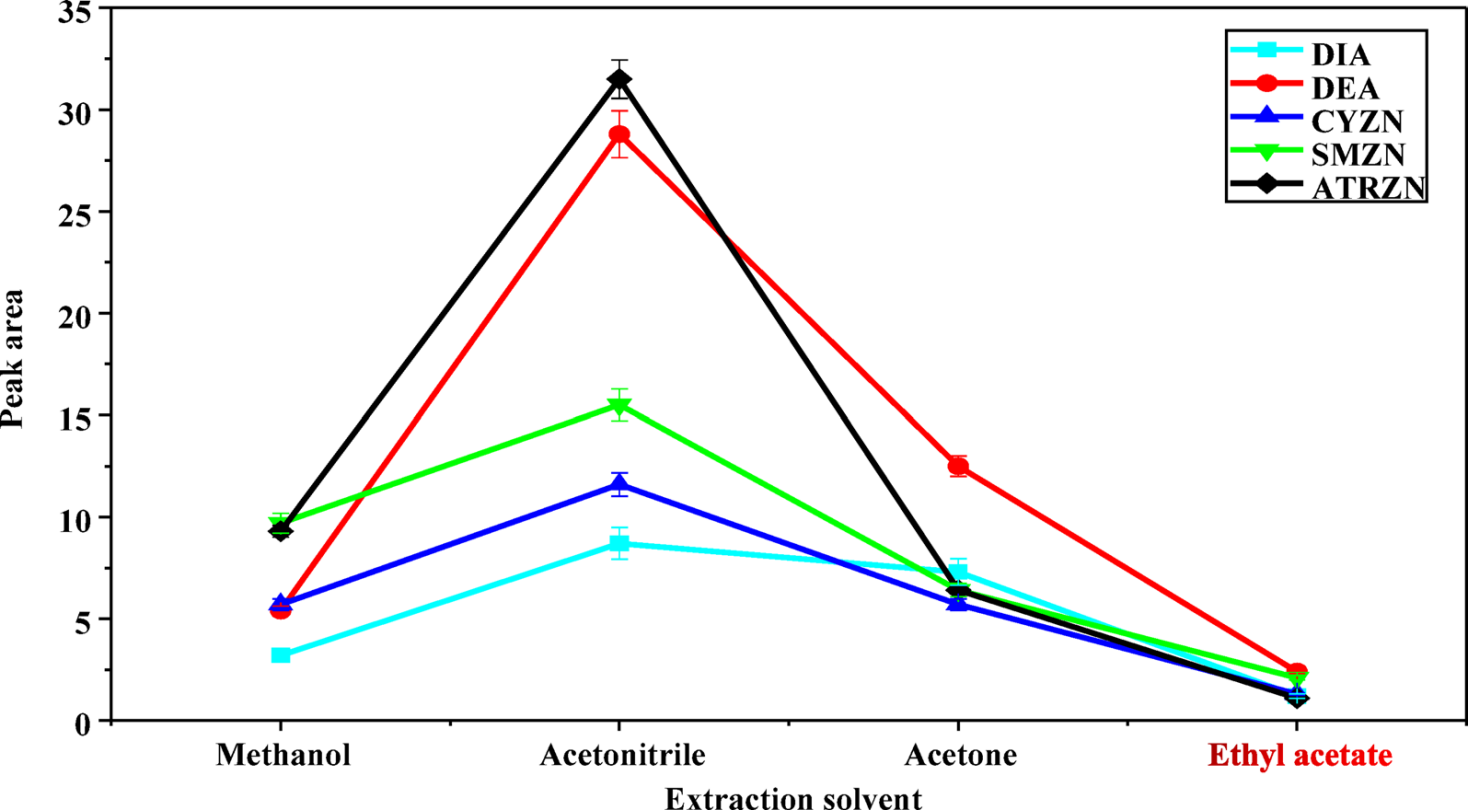
Effect of solvent type on extraction. The extraction conditions: sample amount, 5 mL; amount of salt added, 2 g MgSO_4_; solution pH, 7.0; vortexing time, 1 min; volume of each extraction solvent, 2 mL; centrifugation speed, 4000 rpm for 5 min

Selecting suitable organic solvent for extraction is a crucial step in the SALLE procedures. Ideal organic solvents should possess several key characteristics: a high capacity to dissolve analytes, miscibility with water, simplicity of phase separation upon adding the appropriate salt, and favorable chromatographic behavior [32]. Based on these criteria, four solvents: MeOH, ACN, acetone, and ethyl acetate were evaluated as extraction solvents. A series of experiments were conducted using a 5 mL ultrapure water sample containing 2 g of MgSO_4_ and 2 mL of each organic solvent. As shown in Fig. 1, phase separation was minimal for both methanol and ethyl acetate, a finding that has been reported in the literature [33–35]. The lack of phase separation in methanol may be due to its high polarity, which arises from its hydroxyl group and the hydrogen bonding with water, leading to increased solubility [36]. Figure 1 illustrates the maximum peak area observed when ACN was used as extraction solvent. This can be attributed to ACN’s polarity being closer to that of water, along with its ability to extract ATZN, SMZN, CYZN, DEA, and DIA [37]. Additionally, ACN has lower toxicity compared to other common extraction solvents, aligning with the principles of green chemistry. Therefore, ACN was considered the preferred extraction solvent for this study.

It should be noted that methanol (MeOH), acetonitrile (ACN), acetone, and ethyl acetate are polar, but their polarities are still lower than water. That means, in a water–organic mixture without salts, most of these solvents are completely miscible with water, so no separate phase forms. Although the organic solvents used in this study are partially miscible with water, addition of salts induces phase separation via the salting-out effect [38]. The ions reduce the solubility of the organic solvents in water by binding water molecules, thereby promoting the formation of a distinct organic phase and enhancing extraction of hydrophobic analytes.

#### Effect of the extraction solvent volume

**Fig. 2:**
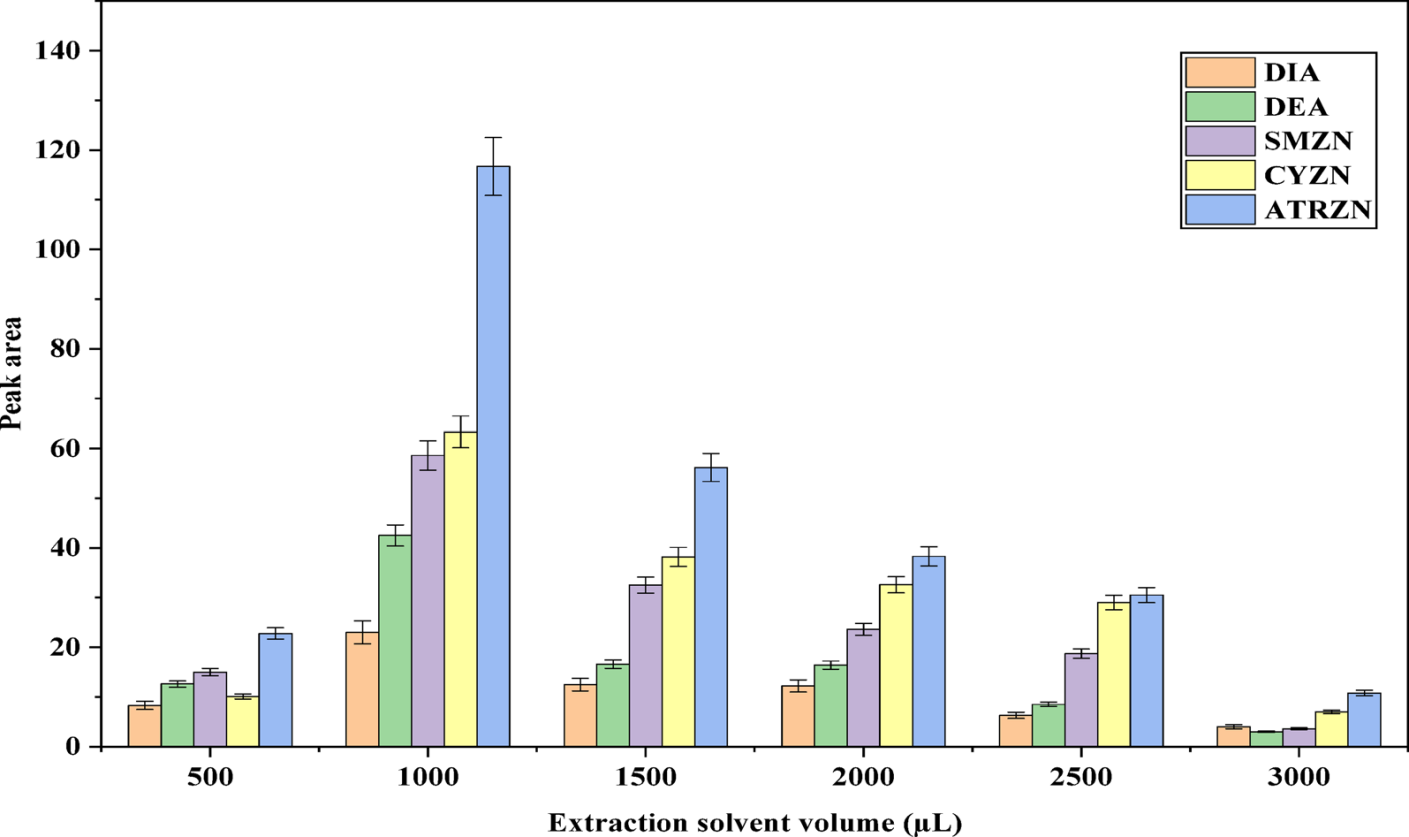
Effect of solvent volume extraction. Extraction conditions: sample amount, 5 mL; extraction solvent, acetonitrile; amount of salt, 2 g MgSO_4_; solution pH, 7; vortex time, 1 min; centrifugation, 4000 rpm for 5 min

The volume of extraction solvent significantly influences the extraction performance of the SALLE technique by affecting the analyte’s solubility in the sample solution [36]. To maximize the enrichment while minimizing environmental toxicity, the extraction solvent volume should be kept as small as possible. However, it must be adequate to effectively extract the target analytes and achieve phase separation ensuring that a sufficient volume of organic phase is collected for subsequent chromatographic analysis [39]. In this study, the volume of ACN was varied from 500 to 3000 µL to assess its effect on extraction efficiency. As shown in Fig. 2, the peak areas for all analytes were increased with ACN volumes from 500 to 1000 µL, but decreased with further increases in ACN volume [34]. At volumes below 500 µL, the interface between the extraction solvent and the aqueous phase was unclear, that makes difficult for collection of the organic layer. The decline in extraction efficiency at volumes above 1000 µL is likely due to a dilution effect from the larger volume. Therefore, 1000 µL of ACN was selected as the optimal volume for all subsequent experiments.

During the extraction of the pesticides analytes, the addition of salts induces the salting-out effect, increasing the ionic strength of the aqueous phase and promoting phase separation between water and organic solvent (acetonitrile). While this improves analyte partitioning into the organic phase, the overall volume of the extraction solvent remains largely unchanged, as the organic layer separates cleanly. This behavior is consistent with the principles of salting-out assisted liquid–liquid extraction (SALLE) [40].

#### Effects of the salt type

**Fig. 3:**
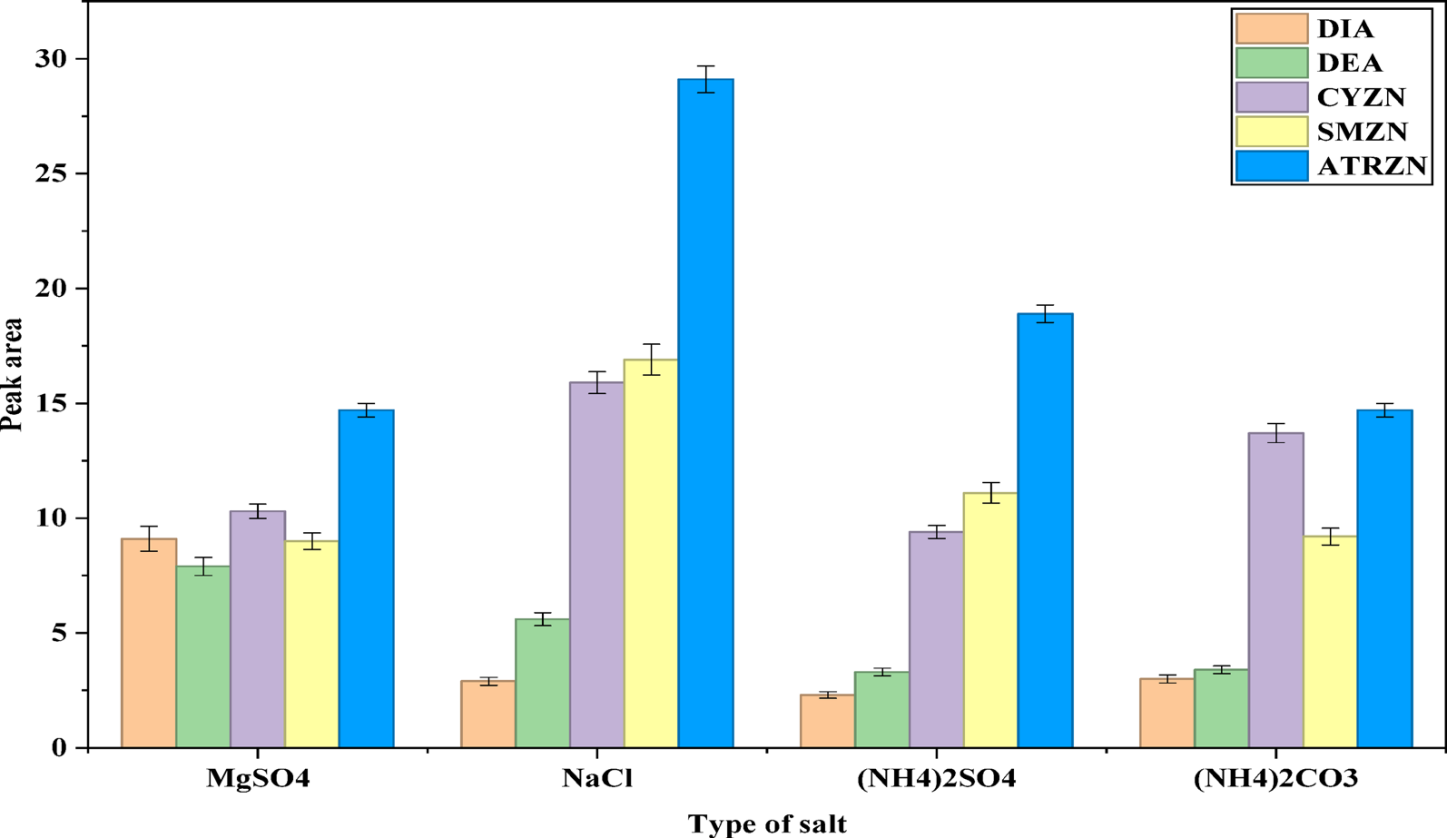
Effect of salt type. Extraction conditions: sample amount, 5 mL; extraction solvent, acetonitrile; volume of extraction solvent, 1000 µL; amount of each salt, 2 g; solution pH, 7.0; centrifugation at 4000 rpm for 5 min; vortex duration, 1 min

The addition of salt can reduce the solubility of both the analytes and the extraction solvent in the aqueous phase, thereby enhancing the transfer of analytes into the organic phase [16, 41]. Different salts can produce varying degrees of phase separation [42]. In this study, the impact of adding 2 g each of potential salting-out agents, including NaCl, MgSO_4_, ((NH_4_)_2_SO_4_, and ((NH_4_)_2_CO_3_) was evaluated. All the salts induce phase separation; however, as illustrated in Fig. 3, the highest instrumental response for all analytes was achieved with NaCl as the salting-out agent. Conversely, MgSO_4_ provided the best instrumental response for atrazine metabolites, most likely due to its high ionic strength per unit concentration in the aqueous phase [43]. Consequently, a mixture of the binary salts (MgSO_4_ and NaCl) was used for the extraction of the analytes in this study.

#### Effect of the ratio and mass of binary salts

**Fig. 4:**
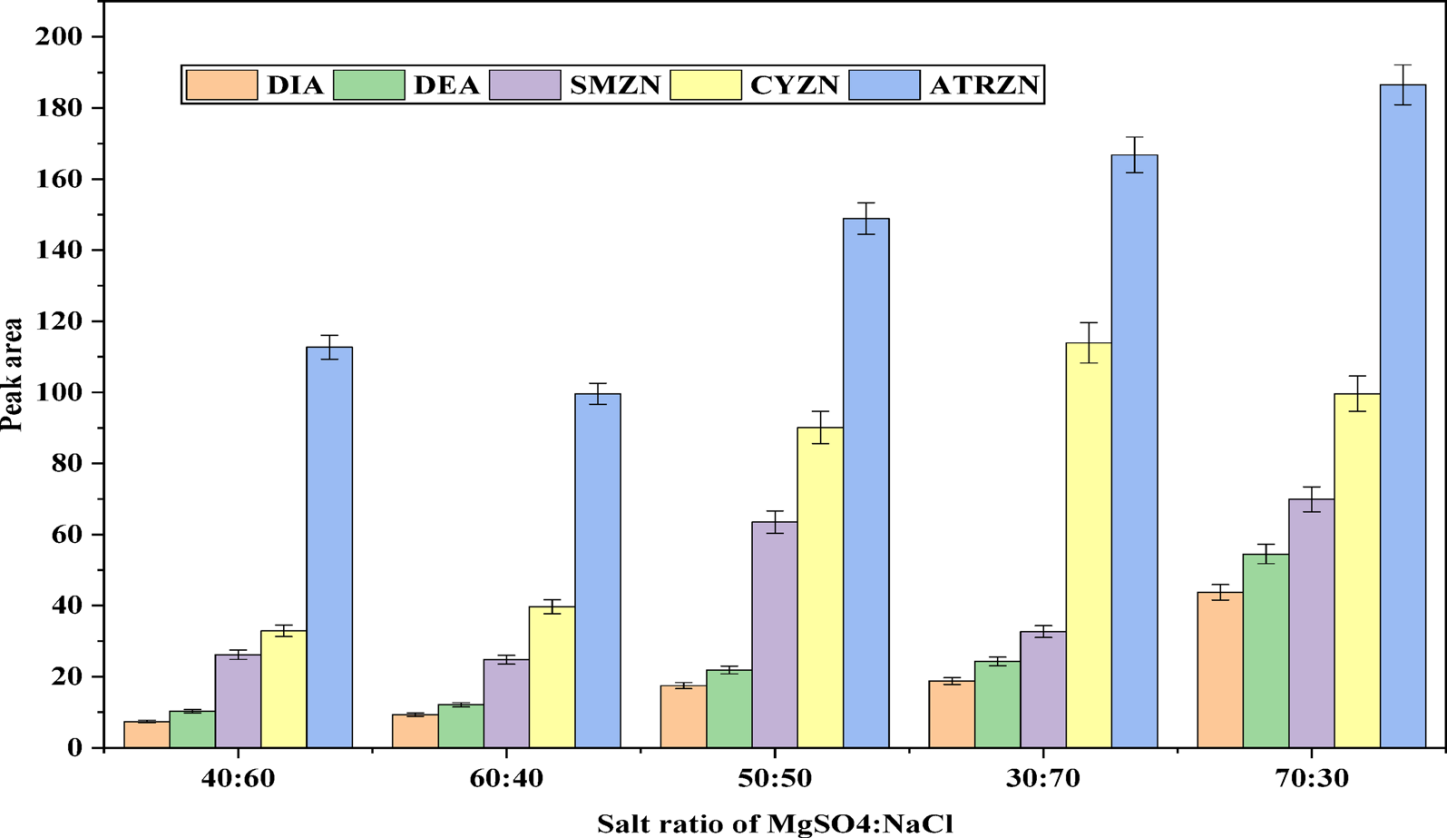
Effect of the salt ratio of MgSO_4_:NaCl . Extraction conditions: sample amount, 5 mL; extraction solvent, acetonitrile; volume of extraction solvent, 1000 µL; salt type, MgSO_4_:NaCl; solution pH, 7.0; centrifugation at 4000 rpm for 5 min; vortex duration,1 min

**Fig. 5:**
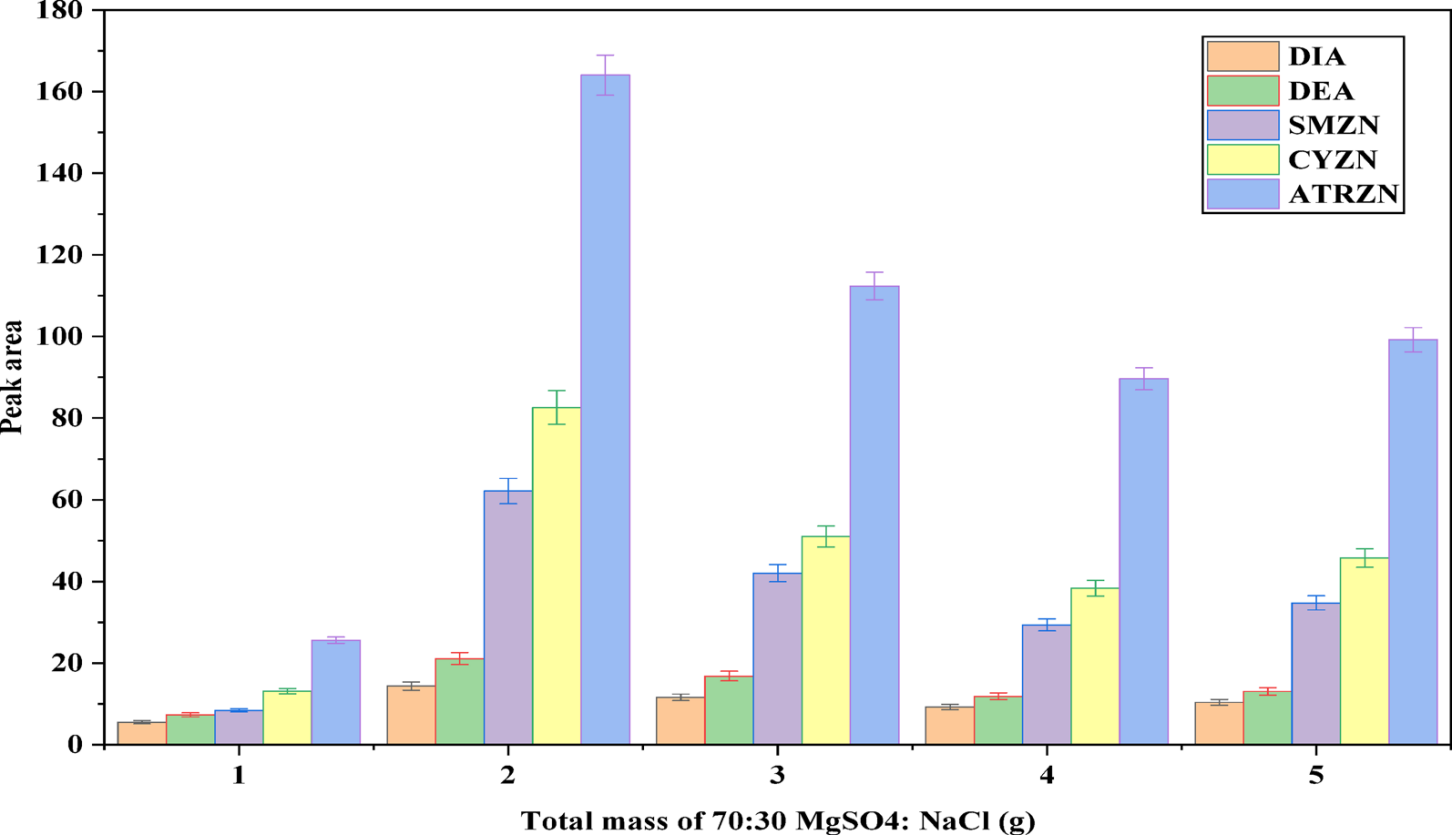
Effect of total mass or amount of MgSO_4_:NaCl ratio. Extraction conditions: sample amoun, 5 mL; extraction solvent, acetonitrile; volume of extraction solvent, 1000 µL; salt type, MgSO_4_:NaCl (70:30); solution pH 7.0; centrifugation at 4000 rpm for 5 min; vortex duration, 1 min

Optimization of the quantity of the two salts, MgSO_4_ and NaCl, is another important parameter. The effect of ratio of the salt quantity was evaluated at 40:60, 60:40, 50:50, 30:70 and 70:30 (MgSO_4_:NaCl) and then 70:30 (MgSO_4_:NaCl) was selected as optimum ratio, as indicated in Fig. 4. Following optimization of the salt quantities, the total mass of salts used in the experiment was assessed. In general, varying the amount of salts can lead to differences in the degree of phase separation [44]. To this end, a salting-out study was conducted by adding varying amounts of MgSO_4_: NaCl ranging from 1 to 5 g, to the aqueous sample solution. As illustrated in Fig. 5, the peak area of the target analytes slightly increased when the total mass of salts rose from 1 to 2 g. However, at higher concentrations, the peaks for all target analytes began to decrease slightly. Consequently, 2 g was selected as the optimal mass for subsequent experiments. Similar findings have been reported to produce an important salting-out effect in the SALLE analytical method, which has been applied to matrices such as fruit juice, yogurt, and carbonated drinks [45].

In other published reports addition of inorganic salts, such as NaCl, was found to facilitate phase separation and has been used for the extraction of pesticides [46]. Similarly, MgSO_4_ is commonly employed to remove polar solvents, particularly water, from nonpolar media [47]. In this study, it was also with this intention that a combination of binary salts composed of NaCl and MgSO_4_ was used to enhance phase separation and recover pesticide residues from spiked fruit juice and water samples.

The selection of NaCl was based on its overall beneficial effect on the extraction efficiency of the parent triazine herbicide compounds used in the study. This enhancement can be attributed to the salting-out effect, which promotes the partitioning of more hydrophobic compounds (higher log K_ow_) into the extraction phase [48]. In contrast, DEA and DIA are more polar metabolites and showed improved extraction efficiency in the presence of MgSO_4_. Therefore, a binary salt system consisting of NaCl and MgSO_4_ was employed to enable efficient extraction of both the parent pesticides (ATRZN, SMZN, and CYZN) and their metabolites (DEA and DIA), benefiting from the increased ionic strength achieved by the combined salts.

#### Effect of sample pH

**Fig. 6:**
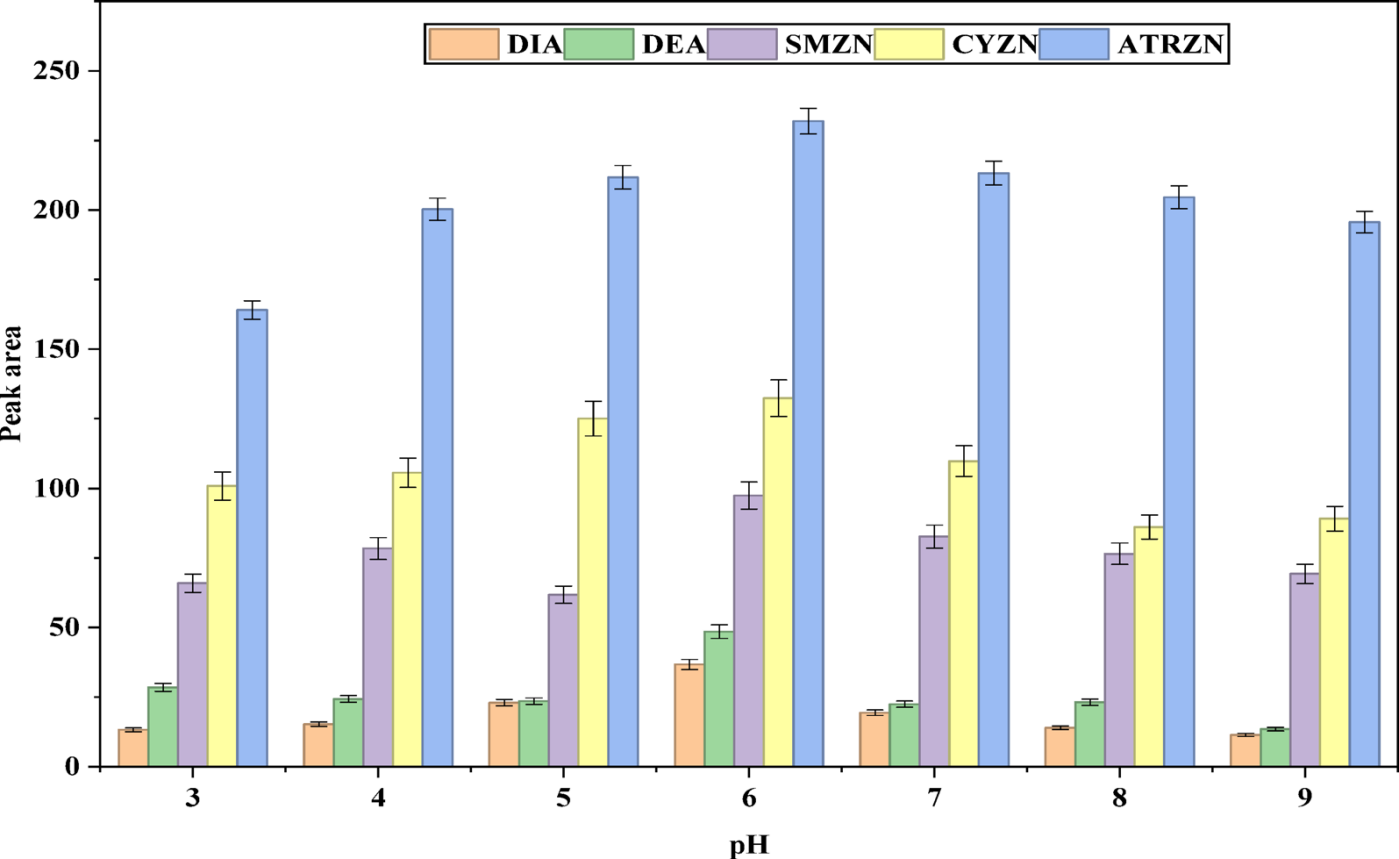
Effect of pH. Extraction conditions: sample amount, 5 mL; extraction solvent, acetonitrile; volume of extraction solvent, 1000 µL; type of salt, 2 g (70:30) of MgSO_4_:NaCl; solution pH, 7.0; centrifugation at 4000 rpm for 5 min; vortex duration, 1 min

In SALLE, the pH of the sample solution significantly influences the extraction efficiency of multi-residue pesticides by influencing their ionization, and solubility in aqueous media [49, 50]. To assess this effect; a series of experiments was conducted, varying the pH from 3.0 to 9.0 in the aqueous solution using HCl and NaOH for adjustments. The results showed that pH 6 is the optimal pH, as demonstrated in Fig. 6. This suggests that the target analytes are more stable in weakly acidic and weakly alkaline conditions, while they may become ionized in strongly acidic or alkaline environments [35]. Consequently, pH 6 was selected as the optimum pH for subsequent experiments.

#### Effect of vortex agitation time

**Fig. 7:**
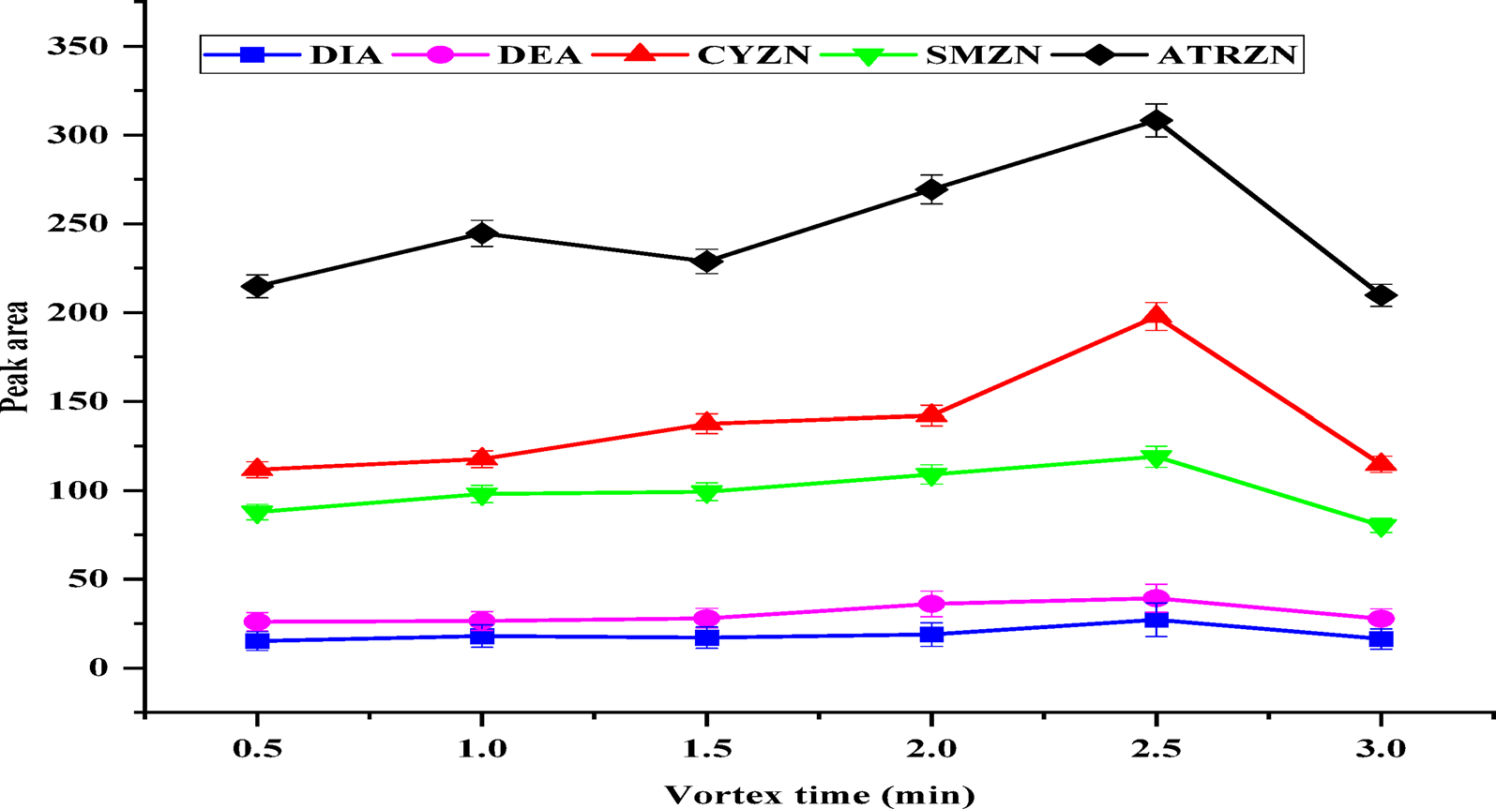
Effect of vortex time. Extraction conditions: sample amount, 5 mL; extraction solvent, acetonitrile; volume of extraction solvent, 1000 µL; type of salt, MgSO_4_:NaCl (70:30); total mass of MgSO_4_:NaCl, 2 g (70:30) added; solution pH, 6.0; centrifugation at 4000 rpm for 5 min; vortex duration, 1 min

Mass transfer is a time-dependent procedure and plays a crucial role in most extraction processes [41]. Vortex agitation was used to enhance the interaction among acetonitrile and the aqueous sample solution, thereby influencing extraction kinetics and promoting the formation of a two-phase system. Additionally, vortex agitation improved the dissolution of the salting-out salt. The optimal vortex time was assessed within the range of 0.5 to 3 min at maximum speed. A slight increase in peak intensity was noted as the vortex time increased from 0.5 to 2.5 min, suggesting that the desperation of pesticides from the sample into the acetonitrile medium occurs relatively quickly. However, extraction efficiency declined after 2.5 min (Fig. 7), likely due to back extraction. Therefore, a vortex time of 2.5 min was chosen for this study.

#### Effect of centrifugation time

**Fig. 8:**
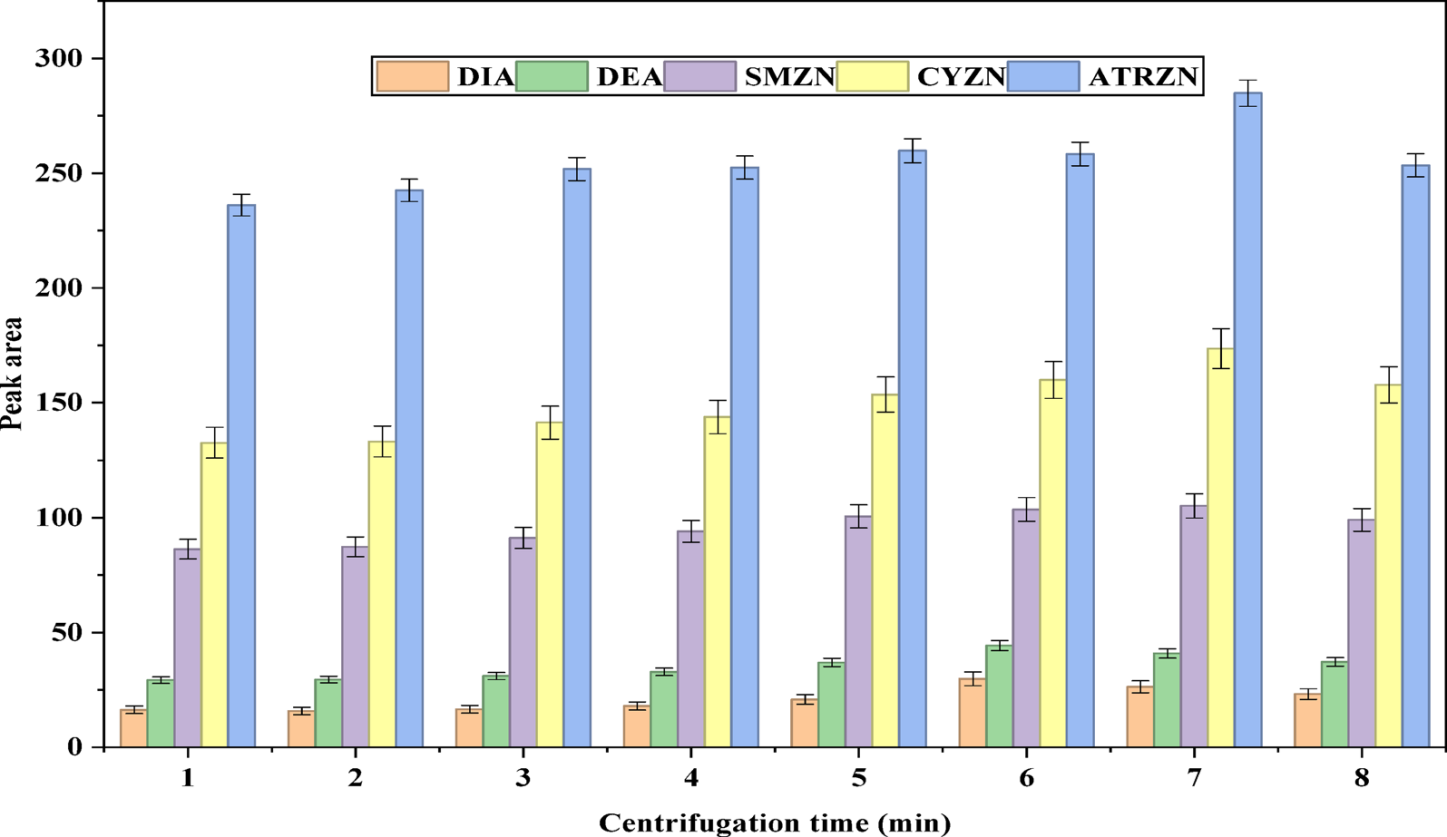
Effect of centrifugation time. Extraction conditions: sample amount, 5 mL; extraction solvent, acetonitrile; volume of extraction solvent, 1000 µL; type of salt, 2 g of MgSO_4_:NaCl (70:30); solution pH, 6.0; centrifugation at 4000 rpm for 5 min; vortex duration, 2.5 min

In SALLE procedures, optimizing the phase separation time is a crucial analytical step for obtaining a clear extract [41]. To determine the optimal conditions, centrifugation times were tested, ranging from 1 to 8 min, in one-minute increments, while keeping the speed constant at 4000 rpm (Fig. 8). Analysis of the peak areas for the target analytes revealed that the highest results were achieved at a centrifugation time of 7 min. Consequently, 7 min was chosen as the optimum centrifugation time for further studies.

#### Analytical performance of the method

The validation of analytical method involved several key parameters: linearity, repeatability, reproducibility, extraction recovery, limit of detection (LOD), and limit of quantitation (LOQ). Linearity was evaluated using both a pure solvent standard calibration curve and matrix-matched calibration. Each curve consisted of six concentrations: 1, 10, 50, 100, 200 and 500 ng/mL, with each concentration measured in triplicate. In contrast, matrix-matched calibration curves were prepared by spiking water and fruit juice samples with target pesticides at various concentrations. The method’s precision was evaluated through both repeatability (intraday precision) and reproducibility (interday precision), with reproducibility measured over three different days. The limits of detection (LODs) and limits of quantitation (LOQs) were calculated using the standard deviation (σ) and the slope of the calibration curve (S). The standard deviation was obtained from nine extraction responses of blank water for each sample type. LODs were determined using the formula 3 × σ/S, while LOQs were calculated with 10 × σ/S [1]. The determined LOD ranged from 0.002 to 0.089 ng/mL, while the LOQ varied from 0.0066 to 0.299 ng/mL. These results confirm the reliability of the developed SALLE method for the selectively separating pesticide residues at trace levels from complex matrices.

Method repeatability (intra-day) and reproducibility (inter-day) were evaluated using ultrapure water spiked at 2, 5, and 10 ng/mL for each analyte. Intraday precision was evaluated by conducting tests on the same day (*n* = 6), while interday precision was assessed across different days (*n* = 9), respectively. For the intraday precision studies, three replicate samples were spiked at each concentration level, extracted, and analyzed in duplicate. Reproducibility was assessed over three consecutive days with same three concentration levels. The results showed that the relative standard deviations (RSDs) ranged from 2.15 to 7.13% for intraday precision and from 3.28 to 8.95% for interday precision, confirming the method’s good repeatability and reproducibility, respectively [51]. The results of analytical method validation are given in Table 2.

**Table 2:** Analytical method validation for the SALLE technique coupled with HPLC-DAD for multi-residue pesticides under study [52]

Analytes	Linear range (ng/mL)	Regression equation	*r* ^2^	LOD (ng/mL)	LOQ (ng/mL)	Repeatability (ng/mL) (%RSD, *n* = 6)			Reproducibility (ng/mL) (%RSD, *n* = 9)		
						2	5	10	2	5	10
DIA	1–500	y = 22.708x + 1.7694	0.991	0.0899	0.299	2.40	2.58	3.96	4.49	3.74	4.61
DEA	1–500	y = 38.837x + 7.2454	0.997	0.0123	0.0408	3.13	3.92	7.13	8.93	3.28	8.85
CYZN	1–500	y = 132.35x + 19.27	0.993	0.0139	0.0463	4.84	5.30	5.92	7.59	6.67	6.23
SMZN	1–500	y = 108.42x + 7.3435	0.995	0.00200	0.00667	5.10	5.81	6.62	8.95	8.06	8.95
ATRZN	1–500	y = 233.59x + 52.664	0.992	0.00325	0.0108	2.15	4.08	3.32	7.51	7.62	7.32

### Application of the method to the real samples

**Fig. 9:**
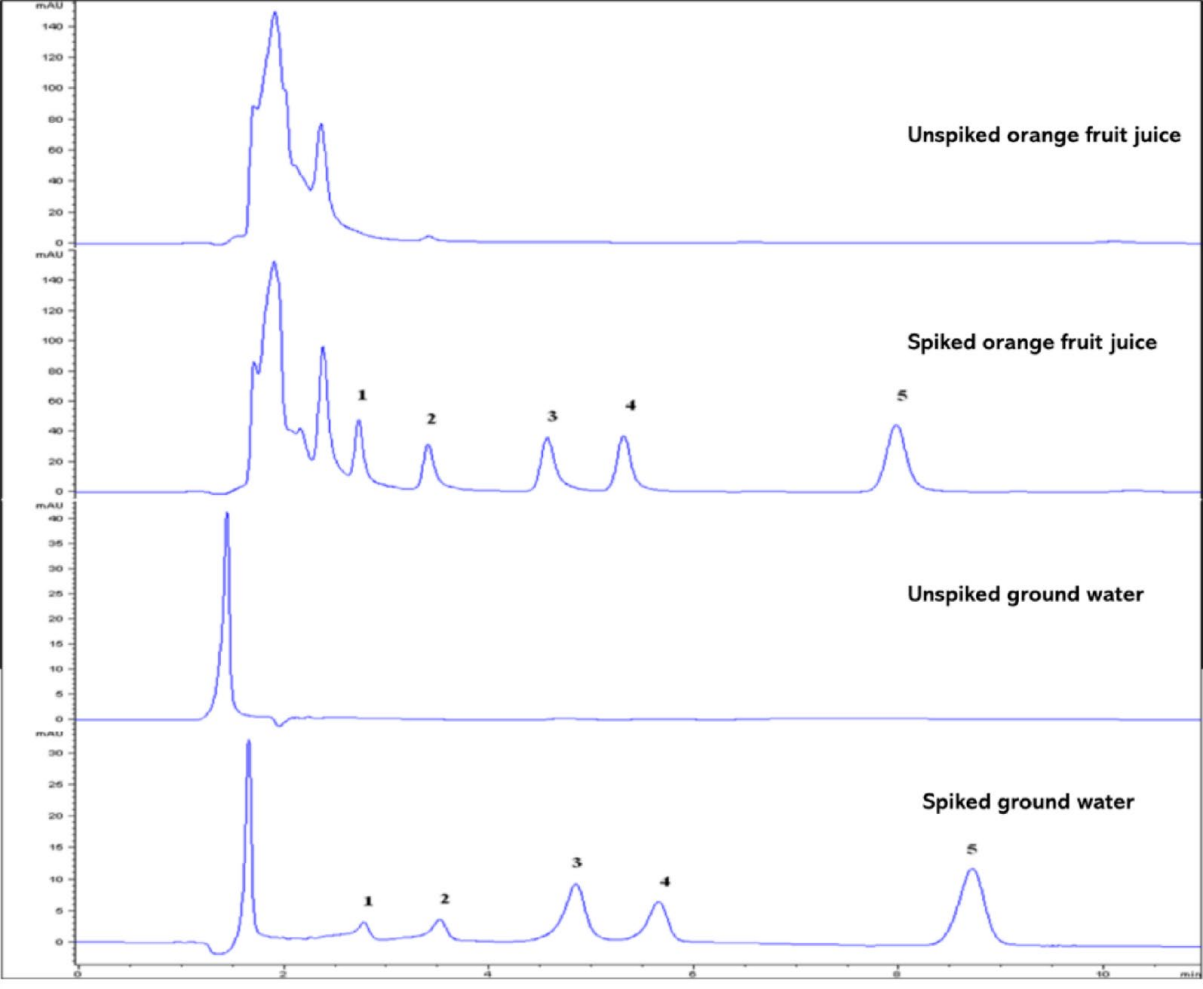
Typical chromatograms of unspiked and spiked orange fruit juice and ground water samples at a concentration level 50 µg L^− 1^. Extraction conditions: sample size, 5 mL; extraction solvent, acetonitrile; extraction solvent volume, 1000 µL; salt type, MgSO_4_:NaCl; amount of MgSO_4_:NaCl added, 2 g; pH of solution, 6.0; centrifugation speed, 4000 rpm for 5 min. Peaks identifications: DIA (1), DEA (2), Cyanazine (3), Simazine (4), Atrazine (5)

In the SALLE method developed in this study, three types of water samples (groundwater, river water and lake water) and three fruit samples (watermelon, orange, and pineapple) were analyzed in order to validate the accuracy of the proposed method. None of the target pesticides were detected in any of the samples, suggesting that either the samples were free from the target pesticides or that their concentrations were below the method’s detection limit. To evaluate the impact of the matrix on the proposed method, the samples were spiked with target pesticides at two concentration levels (2 and 5 ng/mL), which were used for precision studies. The mean relative recoveries (%RR) ranged from 74.06 to 109.90%, with relative standard deviations (RSDs, *n* = 3) below 10% for all samples (Table 3), all results fell within the acceptable range [25]. These findings indicate that the matrices of the real samples did not significantly affect the proposed SALLE method for extracting the target multi-residues pesticides. Representative chromatograms of the water and fruit samples before, and after spiking, along with the corresponding chromatograms of the standard multi-residues, are shown in Fig. 9. These results demonstrate that no observable peaks from interferents or co-extracted compounds appeared at the retention times of the pesticides of interest, confirming that the proposed method is highly selective for analyzing the multi-residues and metabolites of the studied pesticides.

**Table 3:** Relative recovery (RR) values of the proposed method in the fruit juice and water samples

Analyte	Spiked level	Watermelon juice		Pineapple juice		Orange juice		Ground water		River water		Lake water	
		%RR	%RSD	%RR	%RSD	%RR	%RSD	%RR	%RSD	%RR	%RSD	%RR	%RSD
DIA	level 1	107.47	3.82	80.17	4.98	109.53	4.11	95.09	2.40	90.76	4.50	87.90	4.86
	level 2	82.47	4.31	81.53	1.98	76.40	7.30	100.30	2.83	95.40	4.87	89.14	3.77
DEA	level 1	87.23	7.23	109.90	1.50	82.47	4.29	92.35	2.55	86.31	7.09	76.36	2.18
	level 2	85.93	2.44	75.87	5.14	87.33	3.50	101.52	4.10	87.08	5.59	99.50	0.87
CYZN	level 1	86.57	5.04	97.37	4.24	103.93	6.21	82.45	6.11	88.93	1.45	80.94	1.84
	level 2	94.20	4.23	90.00	8.01	94.13	2.14	100.29	3.89	77.21	5.06	74.06	2.31
SMZN	level 1	97.67	3.60	82.10	4.76	96.97	7.45	83.86	7.41	101.34	5.14	92.76	5.00
	level 2	85.93	6.20	97.87	3.57	75.07	5.49	82.44	6.85	84.25	1.38	75.81	4.87
ATRZN	level 1	80.97	1.30	91.00	5.49	98.67	8.74	84.73	1.41	91.53	5.94	88.36	3.39
	level 2	95.73	4.39	100.47	6.89	79.33	4.44	78.76	5.85	76.21	4.78	95.24	4.61

### Comparison of the proposed method with other reported methods

The SALLE method developed in this study for determining multi-residue pesticides in water and fruit samples was evaluated against other reported methods, as detailed in Table 4. This comparison was based on the considered factors such as extraction solvent, linear range, limit of detection (LOD), limit of quantification (LOQ), relative standard deviation (%RSD), recovery (%RR), and extraction time. Notably, the proposed method utilized the relatively less toxic solvent ACN, making it more environmentally friendly compared to other solvents reported in the literature [53–55]. The SALLE method demonstrated a wider dynamic range compared to the findings reported in the literature [53, 54, 56, 57] improved precision, and exhibited shorter extraction time [51, 55] than both traditional SALLE methods and other techniques listed in Table 3. Additionally, it resulted in greater sensitivity compared to the traditional SALLE method [56].

Furthermore, the SALLE method developed was successfully applied for the extraction of multi-residue pesticides from water and fruit samples. The minimal volume of extraction solvent used underscored the method’s environmental sustainability and cost-effectiveness. Additionally, it demonstrated comparable, and in some, instances superior LOD [53–56], LOQ [53, 55], RSD, and %RR values [54–58] when compared to other methods listed in Table 3. This positions the SALLE method as a reliable option for the quantitative and selective extraction and pre-concentration of multi-residue pesticides from the samples and similar matrices. Consequently, the proposed SALLE method can be utilized as a simple, fast, effective, and environmentally friendly alternative for the simultaneous extraction of multi-residue pesticides and their major degradation products from water and fruit, and other samples with similar matrices prior to their quantitative analysis by HPLC–DAD.

**Table 4:** Comparison of the proposed method with other methods applied for the extraction and determination of pesticides in water and fruit juice samples

Methods	Detection	Extraction time (min)	LR (ng/mL)	LOD (ng/mL)	LOQ (ng/mL)	RSD (%)	RR (%)	Ref.
IL-DLLME	HPLC-DAD	2	5–250	1.01–1.57	3.37–5.24	–	70–102.3.3	[53]
CPE	HPLC-UV	30	50–2000	6.79–11.2	–	1.41–5.99	70.5–96.9	[54]
QuEChERS	HPLC-DAD	–	–	20–60	–	1–23	35–131	[55]
SALLE	GC-MS	1	5–5.50.50	0.15–5.50	0.5–5.50	0.2–23	49–93	[56]
DLLME	GC-MS	0.5	2–1000	0.9–5.0.9.0	-	5.1–6.3	86–110	[16]
MW-CPE	GC-MS	–	–	0.003–0.02	0.009–0.052	2–9	46–94	[57]
HS-SPME	GC–MS	45	6.5–56	2.2–10.9	–	6.1–29.5	–	[58]
SALLE	HPLC-DAD	0.5	1–500	0.002–0.089	0.0066–0.299	0.87–8.74	74.06–109.90	This work

## Conclusions

The analytical method combining salting-out assisted liquid-liquid extraction (SALLE), utilizing binary salts, with high-performance liquid chromatography-diode array detection (HPLC-DAD) was developed and successfully applied for the simultaneous determination of three chloro-*s*-triazine herbicide residues along with two of their major degradation products in water and fruit juice samples. This method presents several advantages, including high recovery rates, a broad linear range, short analysis times, and using small amount of organic solvents. The findings indicate that SALLE, paired with a water-miscible extraction solvent (acetonitrile) and a mixture of binary salts of MgSO_4_ and NaCl, as salting-out agents, is a highly reliable extraction and preconcentration technique for ATZN, SMZN, CYZN, DEA, and DIA of multi-residue pesticides and major atrazine metabolites that are challenging human health and natural environments.

The proposed analytical method was found to be suitable to selectively extract ATZN, SMZN, CYZN, DEA, and DIA using binary salt and organic solvents from complex matrices of varying origin. The developed method demonstrated good analytical performances for all the target analytes, as shown by the analytical figure of merits. It was also validated by studying the accuracy and acceptable mean recoveries for the pesticides residues considered. As a result, the SALLE method utilizing binary salts could be used as a selective and sensitive alternative for extraction and preconcentration of multi-residue pesticides and their metabolites from water and fruit samples as well as other trace level pollutants having similar physicochemical properties.

## Data Availability

“Data is provided within the manuscript files”.

## Abbreviations

CPE: Cloud point extraction
DLLME: Dispersive liquid-liquid microextraction
GC-MS: Gas chromatography-mass spectrometry
HF-LPME: Hollow-fiber liquid phase microextraction
HPLC-DAD: High-performance liquid chromatography-diode array detection
HPLC-UV: High-performance liquid chromatography-ultraviolet detection
IL-DLLME: Ionic liquid-based dispersive liquid-liquid microextraction
LLE: Liquid-liquid extraction
LOD: Limit of detection
LOQ: Limit of quantitation
LPME: Liquid-phase microextraction
MW-CPE: Microwave-assisted cloud point extraction
QuEChERS: Quick, Easy, Cheap, Effective, Rugged, and Safe
RR: Relative recovery
RSD: Relative standard deviations
SALLE: Salting-out assisted liquid-liquid extraction
SDME: Single-drop microextraction
SPE: Solid-phase extraction
SPME: Solid phase microextraction
